# Increases in summer serum 25-hydroxyvitamin D (25OHD) concentrations in elderly subjects in São Paulo, Brazil vary with age, gender and ethnicity

**DOI:** 10.1186/1472-6823-10-12

**Published:** 2010-06-14

**Authors:** Sergio S Maeda, Ilda S Kunii, Lilian F Hayashi, Marise Lazaretti-Castro

**Affiliations:** 1 Department of Medicine, Division of Endocrinology, Universidade Federal de São Paulo (UNIFESP) - Escola Paulista de Medicina (EPM), São Paulo, São Paulo, Brazil

## Abstract

**Background:**

Hypovitaminosis D is a common condition among elderly individuals in temperate-climate countries, with a clear seasonal variation on 25 hydroxyvitamin D levels, increasing after summer and decreasing after winter, but there are few data from sunny countries such as Brazil. Many factors can interfere on vitamin D cutaneous synthesis. We aimed at studying the 25OHD variations during winter and summer in an outdoor physically active elderly population living in São Paulo city, and analysed their determining factors.

**Methods:**

Ninety-nine individuals (52 women and 47 men, from 55 to 83 years old) from different ethnic groups were selected from an outdoor physical activity group. Data are reported as Mean ± SD, and we used Pearson Linear Correlation, Student's t-test for non-related samples, Chi-square (χ²) test and One-way ANOVA for analysis.

**Results:**

Mean 25OHD value for the whole group was 78.9 ± 30.9 nmol/L in the winter and 91.6 ± 31.7 nmol/L in the summer (p = 0.005). Mean winter serum 25OHD concentrations were not different between men and women (81.2 ± 30.1 nmol/L vs. 76.7 ± 31.8 nmol/L, respectively), and 19.2% of the individuals showed values < 50 nmol/L. In the summer, we noticed an increase only for men (107.6 ± 31.4 nmol/L) compared to women (76.7 ± 24.0 nmol/L), and 6.5% showed values < 50 nmol/L. A decrease in the mean PTH in the summer compared to the winter was noticed, with PTH levels showing a relationship with 25OHD concentrations only in the winter (r = -0.208, p = 0.041). White individuals showed an increase in mean serum 25OHD in the summer (p = 0.016) which was not noticed for other ethnic groups (Asians, native Brazilians and blacks). An increase in 25OHD values in the summer was observed in the age groups ranging from 51-60 and 61-70 years old (p < 0.05), but not in the age group from 71 years old on.

**Conclusions:**

25OHD values increased during the summer in elderly residents of São Paulo, but to different extents depending on ethnicity, gender and age. This season-dependent increase was noticed only among men, white and who were in the youngest group of individuals.

## Background

Hypovitaminosis D is a common condition among elderly individuals in various temperate-climate countries [1-3]. This vitamin D deficiency causes secondary hyperparathyroidism, which provokes an increase in bone resorption with consequent bone loss and a high fracture risk [4,5]. Studies conducted in low-latitude countries have shown that this is a phenomenon that also occurs in sunny regions, transforming vitamin D deficiency into a world health concern [6-8]. São Paulo is located at 23°34'S and the climate is mild. However, we have noticed very low 25OHD levels in elderly residents of this city, especially among the homebound [9,10]. On the other hand, taking this same elderly population into consideration, there was a high correlation between 25OHD concentrations and the quantity of UVB rays to which the elderly individuals had been exposed throughout the year, suggesting that with an effective sun exposure, even elderly people can produce enough vitamin D at that latitude.

São Paulo is a large metropolis (the biggest city in South America) with over 10 million inhabitants and a high concentration of buildings. During winter months, pollution levels are higher than in summer, a pattern that contributes to the reinforcement of seasonal effects on vitamin D production in the skin. The effects of air pollution were demonstrated in a study done in Belgium where the authors compared the effects of sun exposure on plasmatic 25OHD concentrations in post-menopausal women who lived in the city and in the countryside. This study showed that the women who lived in a large metropolis, such as Brussels, presented lower plasmatic levels of 25OHD (47.07 ± 22.0 nmol/L) than women who lived in the countryside (79.0 ± 22.0 nmol/L) even though the city women were more exposed to the sun. This study demonstrated that 25OHD levels in these two groups of women were inversely correlated with the ozone quantity in those geographic areas. Therefore, urban pollutants, among them the ozone, can diminish the incidence of UVB rays, and consequently compromise the level of skin synthesis of 25OHD [11].

The objective of this study was to prospectively evaluate seasonal variations in serum 25OHD levels and potential interfering factors in an elderly population that participates in outdoor physical activities.

## Methods

The study protocol was previously approved by the Ethics Committee of UNIFESP, and all volunteers gave written informed consent and answered a questionnaire about their living habits (calcium intake, the use of sunscreen and self ethnic identification).

One hundred ten elderly participants living in São Paulo who were regular members of an outdoor physical activity program for elderly people were invited to take part in the study. This physical activity was performed at least twice a week in the mid-morning or afternoon for one hour with little skin coverage. The exclusion criteria were the identification of any relevant laboratory alterations in blood samples.

Two blood samples were taken from each volunteer at different times: one in the winter and another in the summer. One hundred one individuals provided a first blood sample. From those, one volunteer was excluded due to a diagnosis of primary hyperparathyroidism (PTH: 163 pg/mL, Ionized calcium: 1.56 mM and 25OHD: 90.7 nmol/L) and another was excluded because the subject's 25OHD value was too different from the mean group value (280.0 nmol/L). A complete data set was available for 99 subjects (47 men and 52 women) aged between 55 and 83 years old; the mean age was 67.4 ± 6.0 for women and 67.8 ± 4.9 for men. Only ninety-one individuals provided a second blood sample. Based on the questionnaires the volunteers answered about their food and sun exposure habits, only 7 (7%) were taking supplements that contained vitamin D in doses ranging from 200 to 400 IU per day. As the mean of those seven individuals (100.0 ± 40.4 nmol/L) did not differ from the rest of the group (77.2 ± 29.7 nmol/L, p = 0.493), they were included in the group analysis.

### Methods

The calcium ingestion estimate was based on information collected through a summarized questionnaire concerning dairy milk product consumption. The individuals were divided into two groups: lower calcium ingestion (500 mg or less per day) and higher calcium ingestion (more than 500 mg per day). Food calcium content was calculated based on the Food Composition Table of the U.S. Department of Agriculture/Agricultural Research Service, 2001 http://www.nal.usda.gov/fnic/foodcomp/search/.

Blood samples were taken during the months of June 2002 (winter) and December 2002 (summer) after an eight-hour fast. For the first blood sample taken, ionized calcium, total calcium, phosphorus, creatinine, albumin, alkaline phosphatase (AP), 25-hydroxyvitamin D (25OHD), intact parathyroid hormone (PTH), osteocalcin (OC) and carboxyterminal telopeptide of type I collagen (CTX) were measured. In the second sample, 25OHD, PTH, CTX and OC were measured. The serum samples reserved for PTH, OC and CTX were collected in refrigerated tubes and processed in refrigerated centrifuges. All serum samples were collected into refrigerated tubes, processed in refrigerated centrifuges, and frozen at -20ºC until being measured. Ionized calcium was measured after centrifugation.

Total calcium, phosphorus, creatinine and albumin were measured via an automatic colorimetric method (Modular Roche, São Paulo, Brazil). Ionized calcium was measured via a specific ion-electrode method (AVL 9180 Electrolyte Analyzer, Minn). Commercial tests were used to quantify OC and CTX (chemiluminescence - Elecsys analyzers - Roche). AP was measured using a kinetic enzymatic method, and PTH was measured with an in-house electrochemiluminescence immunoassay described elsewhere [12]. Reference values for these methods are presented in Table 1.

**Table 1:** Laboratory and Clinical Variables according to the gender of the volunteers

	Reference Values	Men (n = 47)	Women (n = 52)	P value
**Age (years)**	-	67.8 ± 4.9	67.4 ± 6.0	0.689

**Weight (kg)**	-	74.0 ± 15.0	68.0 ± 14.7	**0.050**

**BMI**	19-25 kg/m^2^	28.1 ± 4.0	27.6 ± 4.7	0.089

**Total Calcium**	8.8-10.2 mg/dL	9.6 ± 0.3	9.8 ± 0.3	**0.030**

**Ionized Calcium**	1.24-1.42 mmol/L	1.31 ± 0.03	1.32 ± 0.04	0.359

**Albumin**	3.2-5.6 g/L	4.1 ± 0.2	4.0 ± 0.2	0.159

**Alkaline Phosphatase**	50-250 U/L	127 [64-268]	140 [115-171]	0.725

**Phosphorus**	2.5-4.5 mg/dL	2.8 ± 0.4	3.3 ± 0.4	**< 0.001**

**Creatinine**	0.4-1.5 mg/dL	1.07 ± 0.12	0.90 ± 0.10	**< 0.010**

**25OHD winter**	> 50.0 nmol/L	81.2 ± 30.1 **(a)**	76.7 ± 31.8	0.472

**25OHD summer**	> 50.0 nmol/L	107.6 ± 31.4 **(a)**	76.7 ± 24.0	**< 0.001**

**PTH winter**	10-55 pg/mL	26.1 ± 15.7 **(b)**	23.6 ± 9.5 **(c)**	0.340

**PTH summer**	10-55 pg/mL	19.1 ± 11.7 **(b)**	14.9 ± 7.6 **(c)**	**0.058**

**CTX winter**	0.01-5.94 ng/mL	0.354 ± 0.160	0.402 ± 0.222	0.222

**CTX summer**	0.01-5.94 ng/mL	0.375 ± 0.162	0.403 ± 0.215	0.485

**OC winter**	11-43 ng/mL	21.0 ± 7.7	21.8 ± 8.4	0.089

**OC summer**	11-43 ng/mL	24.5 ± 10.3	24.2 ± 10.0	0.228

The Student's t-test was used to evaluate differences between men and women (Mean and Standard-Deviation), except for alkaline phosphatase (Mann-Whitney Test, median and variation interval).

P < 0.05 was considered significant. Comparisons represented by letters a, b and c were significantly different (P < 0.001).

25OHD concentrations were determined by an immunoradiometric assay (Nichols Institute Diagnostics, San Juan Capristrano, CA, USA). The intra-assay coefficient of variation was 4.8%, and the inter-assay coefficient of variation was 16.0% for the lowest values (mean: 35.5 nM) and 3.0% for the highest control (mean: 154.0 nM). Hypovitaminosis D was considered for 25OHD concentrations lower than 50 nmol/L.

### Statistical Analysis

The Student's t-test for non-related samples was used to evaluate the differences between men and women; the chi-square (χ²) test was used to evaluate the proportion of the use of sunscreen between the genders, and One-Way ANOVA test was used to test for differences between two independent groups depending on the season. Correlations between 25OHD and the other quantitative variables were evaluated through the Pearson Linear Correlation. Data are reported as Mean ± SD (Standard Deviation). Differences were considered significant if P < 0.05.

## Results

The final sample was composed of ninety-nine individuals, 47 men (47.5%) and 52 women (52.5%), aged between 55 and 83 years old (mean: 67.4 ± 6.0 years for women and 67.8 ± 4.9 years for men). As for the ethnic origin, the sample was composed of 60 whites (60.6%), 19 Asians (19.2%), 8 native Brazilians (8.1%) and 12 Blacks (Afro-descendents) (12.1%).

The results were obtained for the group as a whole and were separated by gender in relation to laboratory parameters as described in Table 1. Men weighed more than women. Women presented the highest total calcium and phosphorus concentrations. Statistic differences between genders were not found for the following parameters: age, body mass index (BMI), ionized calcium, albumin, CTX and OC.

**Figure 1:**
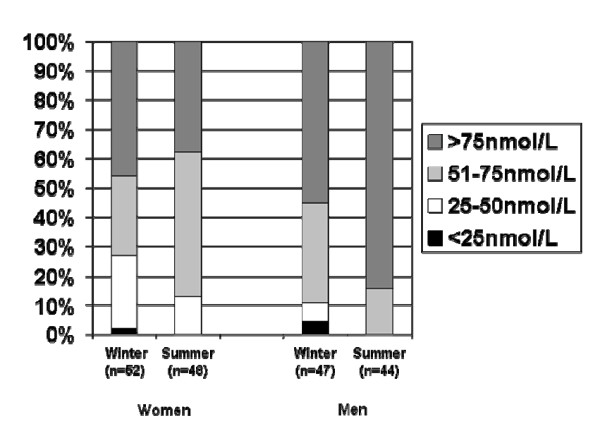
**Distribution of 25OHD (nmol/L) concentrations according to gender and season**.

The mean serum 25OHD of the whole group in the winter was 78.9 ± 30.9 nmol/L, without any statistical difference between men and women (81.2 ± 30.1 nmol/L vs. 76.7 ± 31.8 nmol/L, respectively, p = 0.472). In the summer, the mean of the whole group was 91.6 ± 31.7 nmol/L, and only men presented a significant increase in 25OHD levels (107.6 ± 31.4 nmol/L, p < 0.001); consequently, their mean value was higher than that of the women during this season (76.7 ± 24.0 nmol/L, p < 0.001, Table 1). In the samples taken in the winter, 2% of the women and 4.3% of the men showed values < 25 nmol/L, and 25% of the women and 6.4% of the men presented values between 25 and 50 nmol/L. However, in the summer, values < 25 nmol/L were not found in any of the individuals from either gender. On this same occasion, values between 25 and 50 nmol/L were observed in 12.5% of the women and in none of the men (Figure 1). There was only one case of secondary hyperparathyroidism detected in the winter (PTH: 81.0 pg/mL, ionized calcium: 1.28 mM, creatinine: 1.5 mg/dL), which normalized in the sample taken in the summer.

Mean serum PTH decreased in the summer for men and women; even though they were not different between themselves in the summer and in the winter. The bone remodelling bone markers (CTX and OC) did not differ by gender or by season (Table 1).

Forty nine individuals (49.5%) were classified as lower calcium intakers and 50 individuals (50.5%) had higher calcium intake. There was no difference in 25OHD (p = 0.449 in the winter and p = 0.124 in the summer) or PTH (p = 0.654 in the winter and p = 0.812 in the summer) concentrations between higher and lower calcium intakers.

Twenty seven women (51.9%) and only 3 men (6.4%) declared to use sunscreen (protection factor between 20 and 50) on the face and arms, even in the winter. There was no difference between 25OHD levels from sunscreen users and non-users (77.3 ± 29.6 nmol/L and 84.3 ± 32.9 nmol/L, p = 0.117, respectively). Even among the women who did not use sunscreen, we did not notice any increase in 25OHD in the summer (winter: 72.8 ± 30.6 nmol/L and summer: 79.50 ± 29.7 nmol/L, p = 0.449). PTH levels significantly decreased in the groups as a whole, regardless the use (n = 30, 30.3%) or non-use (n = 69; 69.7%) of sunscreen (p = 0.024 and p < 0.001, respectively). Nevertheless, PTH levels were not different between the two groups (p = 0.143 in the winter and p = 0.352 in the summer) in any season. There were no differences on bone remodelling markers among the individuals who used or not sunscreen during both seasons.

We found a significant correlation between 25OHD and PTH in winter (r = -0.208, 95% CI -0.39; -0.009, p = 0.04). No correlations were found between 25OHD and CTX, OC, ionized calcium, total calcium, albumin, BMI, phosphorus, alkaline phosphatase or creatinine.

The proportions of men and women in the various ethnic groups were not different (p = 0.437). Only white individuals presented a significant summer increase in 25OHD levels (p = 0.016) (Table 2). Whites and Natives, but not blacks and Asians, presented a significant decrease in PTH levels in the summer in comparison to the winter levels (p < 0.001 and p = 0.027, respectively) (Table 2). Mean serum CTX and osteocalcin did not vary between winter and summer in any ethnic groups.

**Table 2:** Distribution of 25OHD (nmol/L) and PTH (pg/mL) concentrations according to ethnicity and the season in which the sample was taken

*Ethnicity and season*	*25OHD winter*	*25OHD summer*	*P value*	*PTH winter*	*PTH summer*	*P value*
Whites (n = 60)	77.9 ± 28.5	92.5 ± 32.2	**0.016**	24.9 ± 12.3	16.9 ± 10.3	**< 0.01**
Asians (n = 19)	86.7 ± 40.9	97.7 ± 36.9	0.280	22.9 ± 10.3	16.0 ± 8.5	0.073
Blacks (n = 12)	70.2 ± 14.8	79.1 ± 21.7	0.488	21.9 ± 9.6	17.0 ± 6.6	0.299
Natives (n = 08)	80.3 ± 39.0	89.6 ± 27.3	0.567	33.7 ± 23.9	19.8 ± 16.0	**0.027**

ANOVA with repeated measuring was used (significant when P < 0.05)

**Figure 2:**
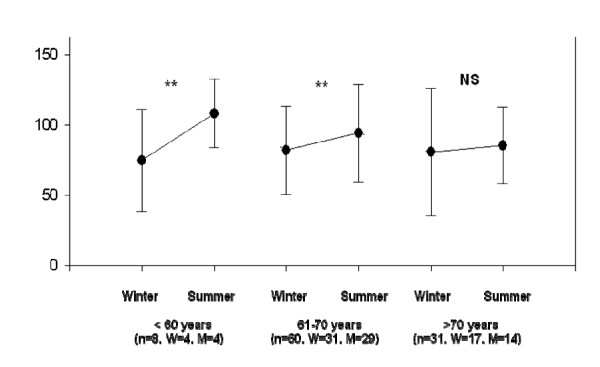
Seasonal variations in 25OHD (nmol/L) concentrations according to age ** p < 0.05 and NS: not significant, W = women, M = men.

When the individuals were divided into three groups according to age, a significant increase in 25OHD concentrations was noticed in the summer in the groups aged between 51-60 years old (n = 8; in the winter: 74.8 ± 34.0 nmol/L; in the summer: 108.2 ± 22.6 nmol/L, p < 0.05) and between 61-70 years (n = 60; in the winter: 81.8 ± 31.3 nmol/L; in the summer: 94.2 ± 34.6 nmol/L, p < 0.05). However, there was no significant variation in the group aged 71 and over (n = 31; in the winter: 80.7 ± 44.8 nmol/L, and, in the summer: 85.4 ± 26.8 nmol/L) (Figure 2). The proportions between women and men in those groups were not different (chi-square test, p = 0.199).

## Discussion

Our results indicate the existence of a seasonal variation on 25OHD concentrations in the individuals who took part in this study; this variation was influenced by factors such as gender, ethnicity and age. We had previously demonstrated the existence of a seasonal variation in healthy young individuals who also lived in São Paulo city [13]. Those individuals were aged between 17 and 33 years old, and they had mean serum 25OHD of 62.9 ± 23.5 nmol/L in the winter which significantly increased to 97.8 ± 33.5 nmol/L during summer. In the meantime, we demonstrated in another study a very high prevalence of lower levels of 25OHD (< 50 nmol/L) among the elderly living in the community whose mean value was 49.5 ± 28.5 nmol/L. Even higher percentage of hypovitaminosis D (71.2%) was found among the elderly in nursing homes (mean values: 36.5 ± 23.5 nmol/L), as well as a high prevalence of secondary hyperparathyroidism in both groups (54.0% and 61.7%, respectively) (9-10). However, the prevalence of hypovitaminosis D in this group of individuals who practice outdoor physical activities was much lower, which reinforces the importance of sun exposure and lifestyle even for older people. The percentage of elderly individuals with hypovitaminosis D was 19.2% in the winter and 6.5% in the summer.

Carnevale *et al*. demonstrated differences in 25OHD levels between genders, with men presenting higher values than women in the winter (mean values: 51.2 ± 13.5 vs. 38.0 ± 14.2 nmol/L, respectively) as well as in the summer (mean values: 97.5 ± 21.0 vs. 76.7 ± 20.0 nmol/L, respectively) [14]. Dawson-Hughes *et al.*, also found significant differences according to gender in an elderly population from Boston, whose mean age was around 70 years old [15]. Men had higher vitamin D concentrations when compared to women (82.4 ± 35.8 vs. 68.9 ± 32.1 nmol/L, respectively); this difference was evident only in the summer. In our study, a 25OHD seasonal increase was detected only among men but not among women, who represent the highest risk group for osteoporosis. Women used sunscreen more often than men, but the use of sunscreen cannot be responsible for the lack of increase in 25OHD because there was not a significant difference between women who used it and women who did not. This result suggests that there are other factors involved.

A PTH decrease was observed in men and in women in the summer, but the 25OHD increase was only significant among men. Even though there was no increase in 25OHD values among women, the proportion of those who had hypovitaminosis D decreased significantly (Figure 1), which can explain the significant PTH reduction observed in this group in the summer. Only one patient had secondary hyperparathyroidism (1%). This low prevalence could be the reason why we did not find correlations between CTX and OC with PTH, as the increase in those markers has been correlated with secondary hyperparathyroidism [16].

Serum 25OHD levels were inversely correlated with PTH (r = -0.208; p = 0.041) only in the winter. This low correlation between 25OHD and PTH is similar to that described by other authors [1,4,17-21]. In the summer, however, this correlation disappeared, probably, because the higher 25OHD concentrations stopped interfering with PTH secretion. The relationship between PTH seasonal variations and bone mass and fracture risk is already well defined [22].

There were only few participants taking vitamin D supplements and their 25OHD levels did not differ from the rest of the group, showing that the given doses (between 200 and 400 IU/day) were insufficient to raise the 25OHD concentrations.

In our study, we observed that only the white individuals showed an increase in 25OHD concentrations in the summer. Those seasonal changes in 25OHD were not observed in other ethnic groups and confirm the importance of melanin content on the rate of skin synthesis of 25OHD. Some authors showed that black individuals had higher PTH concentrations and lower 25OHD concentrations [23,24]. Others showed that blacks have the same capacity as Caucasians to produce 25OHD but require a much larger dose of ultraviolet radiation [25]. This aspect becomes very important in countries like Brazil, where the miscegenation index among races, mainly blacks and whites, is really high. According to the 2000 Brazilian Demographic Census, whites represent 53.7% of the population, whereas brown (mixed-race) people and blacks represent 44.7% (data from IBGE - Instituto Brasileiro de Geografia e Estatística). In São Paulo State, however, according to data from the same census, the white population represents 70.7%, and the brown and black populations represent 27.2% of the total. In our study, the percentages of whites and blacks were close to those proportions, but this data cannot be generalized to the whole Brazilian population.

The 25OHD synthesis declines with aging probably because the skin thickness tapers down and the cutaneous 7-dehydrocholesterol content is reduced [26,27]. The age-related changes in the skin occur mainly in the dermis layer. The entire elastic fiber strucuture in the papillary dermis shrinks and sags, and the number of superficial capillary loops and tufts in the papillary body just beneath the epidermis is markedly reduced [28]. A lower increase in 25OHD concentrations in the oldest group was detected in the summer (Figure 2). Even so, the oldest individuals (70 years old and over), evaluated in this group during the winter, presented 25OHD concentrations more adequate (74.3 ± 27.9 nmol/L) than free-living elderly patients (49.5 ± 28.5 nmol/L) and homebound residents (36.5 ± 23.5 nmol/L) living in the same city [9,10]. This suggests that the quantity of UVB radiation at this latitude is enough for an adequate skin production, even for elderly people, once they get the minimum necessary. When this exposure does not occur, our findings reinforce the need for oral supplementation for elderly people, mainly in the winter [11,29,30].

The lack of a more accurate data about the quantity of sun exposure and about the use of sunscreen, including mean corporeal surface area exposed, and a more detailed evaluation of vitamin D intake from the diet, were, in our point of view, was the main limitation of the study. However, it is already known that vitamin D content in a daily Brazilin diet is usually very low [140 IU/day or 3.5 (3.0-3.9) mcg/day] [31]. The ethnic distribution, even though far from the composition of the Brazilian population, was as a whole, somewhat representative of the region where the study took place. Furthermore, we had a small number of blacks and natives, which could have skewed the evaluation performed according to ethnicity because, ideally, for this type of analysis, we would need a larger representation from those ethnicities.

Some authors have considered the cut-off value for the definition of Vitamin D insufficiency as being 50.0 nmol/L because at values lower than this, there is a bone resorption associated with a PTH increase [32,33]. Others however consider values of 70.0-80.0 nmol/L as the minimum accepted level for fracture prevention and maximum calcium absorption. Our study revealed a mean winter 25OHD value of 78.9 ± 30.9 nmol/L, but the evidenced correlation with PTH at that moment suggests that the ideal concentrations should be higher. Indeed, this correlation was lost in summer, when the mean serum value was 91.6 ± 31.7 nmol/L. These data agree with Vieth *et al*., who defend the idea that the minimum values for elderly people should be around 100 nmol/L [4,32,33].

## Conclusions

We conclude that the elderly residents of São Paulo city (23°34'S) who regularly practice outdoor physical activities regularly have, in general, a mean serum 25OHD within an acceptable level and very similar to the values we found in young healthy individuals who live in the same city [13]. Confirming these data, a lower prevalence of secondary hyperparathyroidism was found. The serum 25OHD concentrations increased in the summer in the whole group, but in a different way concerning gender, age and ethnicity. This increase could be noticed only among men, white and in the youngest group of individuals. According to our data, the most probable group for hypovitaminosis D would be elderly women over 70 years old and individuals with darker skin for whom the supplementation of cholecalciferol would be recommended to avoid the seasonal 25OHD oscillation. Furthermore, sun exposure should be encouraged for elderly people because our data suggest that, at least in 23°S latitude; they are able to synthesize vitamin D in their skin.

## Abbreviations

25OHD: 25 hidroxyvitamin D; PTH: Parathyroid hormone; UVB: Ultraviolet radiation B; UNIFESP: Universidade Federal de São Paulo (Federal University of São Paulo); OC: Osteocalcin; AP: Alkaline phosphatase; CTX: Carboxyterminal telopeptide of type I collagen; IBGE: Instituto Brasileiro de Geografia e Estatística (Brazilian Institute of Geography and Statistics)

## Competing interests

The authors declare that they have no competing interests.

## Authors' contributions

SSM - main author. ISK - carried out the immunoassays. LFH - carried out the immunoassays. ML-C - conceived of the study, and participated in its design and coordination and helped to draft the manuscript. All authors read and approved the final manuscript

## Authors' information

SSM - M.D. at the UNIFESP (Bone Metabolism section), and Professor at the Santa Casa de São Paulo Medical School

ML-C - M.D., Ph.D. and Professor at the UNIFESP (Bone Metabolism section)

## Pre-publication history

The pre-publication history for this paper can be accessed here:

http://www.biomedcentral.com/1472-6823/10/12/prepub
